# Black-and-White Ruffed Lemur (*Varecia variegata*) in Captivity: Analysis of the Oral Microbiota in a One Health Perspective

**DOI:** 10.3390/ani11102905

**Published:** 2021-10-08

**Authors:** Carolina Silva, João F. Requicha, José J. Martins, Aida Duarte, Isabel R. Dias, Carlos A. Viegas, Maria J. Saavedra

**Affiliations:** 1 Department of Veterinary Sciences, University of Trás-os-Montes e Alto Douro, 5000-801 Vila Real, Portugal; carolinalogssilva@gmail.com (C.S.); jfrequicha@utad.pt (J.F.R.); idias@utad.pt (I.R.D.); cviegas@utad.pt (C.A.V.); 2 Animal and Veterinary Research Center (CECAV) and AL4AnimalS, University of Trás-os-Montes e Alto Douro, 5000-801 Vila Real, Portugal; jjulio@utad.pt; 3 Department of Animal Science, University of Trás-os-Montes e Alto Douro, 5000-801 Vila Real, Portugal; 4 Interdisciplinary Centre of Marine and Environmental Research (CIIMAR), Terminal de Cruzeiros do Porto de Leixões, 4450-208 Matosinhos, Portugal; 5 Department of Microbiology and Immunology, Faculty of Pharmacy and Research, University of Lisbon, 1640-042 Lisbon, Portugal; aduarte@ff.ul.pt; 6 Center of Egas Moniz (CiiEM), 2825-001 Monte da Caparica, Portugal; 7 Center for the Research and Technology of Agro-Environmental and Biological Sciences (CITAB) and Inov4Agro, University of Trás-os-Montes e Alto Douro, 5000-801 Vila Real, Portugal

**Keywords:** one health, lemurs, multidrug-resistant bacteria, oral microbiota, antibiotics, biofilm

## Abstract

**Simple Summary:**

The emergence of multidrug-resistant bacteria is a serious public health problem. Wild animals are known to be sources of multidrug-resistant bacteria as well as infectious diseases, some of them transmissible to humans. Due to many factors, such as the destruction of natural habitats or climate change, contact between wild animal species and humans is increasing. Thus, it is particularly important that studies be carried out in wildlife species to assess the possible existence of multidrug-resistant bacteria. In this case, the chosen species was the black-and-white ruffed lemur belonging to a zoo. Through a sample of the oral cavity, it was possible to know that half of the bacteria isolated in this group of animals were resistant to, at least, one antibiotic and one of them is resistant to an antibiotic for exclusive use in a hospital environment. Captive wild mammals can be a source of multidrug-resistant bacteria and studies such as this could contribute to the development of strategies to prevent the spread of this public health program.

**Abstract:**

This study aimed to characterize the susceptibility profile to antibiotics and biofilm formation of Gram-negative bacterial isolates obtained from the oral cavity of the black-and-white ruffed lemur (*Varecia variegata*). From eight individuals from a zoo located in Portugal, samples of the oral microbiota were collected with sterile swabs and then placed in closed tubes with a transport medium. Culture was carried out for media of Gram-negative bacteria. Twenty-two isolates were obtained and subjected to susceptibility tests to twenty-five antimicrobial agents belonging to seven different classes. All tested isolates demonstrated resistance to, at least, one antibiotic, and it was possible to observe multidrug resistance in 11 of the 22 isolates (50%). It should be noted that an isolate showed phenotypic resistance to imipenem, an antibiotic for exclusive use in a hospital environment. All the isolates showed an increasing ability of biofilm formation over time. The obtained results show that wild mammals in captivity could be reservoirs and potential sources of multi-resistant pathogens. In view of this fact and considering the One Health concept, it will be advisable to establish local monitoring programs worldwide that benefit and protect human, animal and environmental health.

## 1. Introduction

The black-and-white ruffed lemur (*Varecia variegata*) is a small primate from the southeastern rainforests of Madagascar [1]. It is a species ranging from 3.5 to 4.6 kg [2], with diurnal habits and is mostly frugivorous [3,4,5]. They live in the form of socially stable “communities”, with the aim of defending their food resources [6]. They are a seasonal species [7,8,9,10,11,12] that usually generates litters of two to three offspring [10,11,13]. Due to the fact that it is considered an endangered species, many zoological parks house these lemurs, integrated in wildlife conservation programs. However, the surroundings and resources are different from those found in wildlife, despite all efforts being made to make the environment as close as possible to its geographic area of origin. There will always be some degree of possible contact with humans, especially with the personnel in charge of their management and feeding.

Climate change, destruction of natural habitats, and globalization lead to an ever-closer contact between humans and wildlife animals. In recent years, an increase in scientific publications on wildlife and public health issues alerts us to the fact that wildlife animals can host several emerging infectious diseases [14] or multidrug-resistant bacteria. The concept of antibiotic resistance is a complex public health issue, in which our ability to treat bacterial infections could be seriously compromised [15,16], and which is characterized by complex interactions among microbial populations that affect human and animal health, and the environment [17].

Thus, it is imperative to take into account the concept “One Medicine, One Health, One World”, in which it is important that measures are taken to ensure the effectiveness of antibiotics currently available [17]. When relating these different themes, there is a need to research, promote and improve surveillance in the use of antibiotics through the evaluation of the spread of multi-resistant bacteria in different ecological niches, combined with the study of species considered to be in danger of extinction. Moreover, according to the World Organisation for Animal Health, oral health is an important element of general health and is crucial for animal welfare and quality of life.

Previously, the intestinal microbiota of the black-and-white ruffed lemur had been investigated and characterized, including a focus on antibiotic resistance [18,19]. However, the characterization of its oral microbiota, associated with a profile of susceptibility to antibiotics, has never been carried out. Thus, the objective of this study was to monitor the antibiotic-resistant profile of Gram-negative bacterial isolates obtained from the oral cavity of captive black-and-white ruffed lemurs, in view to understand the role that animals living in captivity can play as possible sources of multi-resistant bacteria.

## 2. Materials and Methods

### 2.1. Animals

Eight black-and-white ruffed lemurs (*Varecia variegata*), two males and six females, between 3 and 8 years old, from a zoological park located in Northern Portugal were selected for this study. This zoological park houses three hundred animals, among which and in greater number, mammals, followed by birds and finally reptiles. This group of lemurs, originally founded with two individuals, has been housed in the zoological park for more than 10 years. Due to the origin of these animals, high inbreeding within the group cannot be excluded. Lemur facilities were created specifically for this species, allowing the animals to explore the space in a three-dimensional way. It has an area with dense vegetation which allows the animals to take refuge when they wish, and another area, more open, with equipment such as trunks, branches, climbing ropes, and wooden shelters.

### 2.2. Sample Processing and Isolation

Under the scope of a collaboration protocol between the zoological park and the University of Trás-os-Montes and Alto Douro, a specialized panel composed of a veterinary physician and two authorized persons to perform animal experimentation, with accreditation number 020/08 by the Federation of European Laboratory Animal Science Associations (FELASA) performed a thorough clinical examination of the eight individuals. During this routine check-up, it was possible to collect sterile swabs from the four quadrants of the oral cavity, only by manual animal restraining with no need for sedation. The entire procedure was conducted in accordance with the European Animal Welfare Directives (Directive 98/58/CE and Decreto-lei no 64/2000).

The samples of the oral microbiota were placed in closed tubes with a transport medium and properly identified. A culture was carried out for media of Gram-negative bacteria according to the methodologies implemented in the Microbiology Laboratory of the University of Trás-os-Montes and Alto Douro and the Faculty of Pharmacy of the University of Lisbon. The media used, without and with antibiotics, were Chromocult^®^ Coliform Agar and MacConkey Agar following the manufacturers’ instructions. Twenty-two isolates were obtained and those with a greater diversity of colonies (size, colour, or number) were selected to susceptibility tests for twenty-five antimicrobial agents, representative of seven different groups of antibiotics, following the European Committee on Antimicrobial Susceptibility Testing’s (EUCAST) standards using the disk diffusion method.

### 2.3. Identification of Isolates

All isolates were identified by standard biochemical methods (indole, Voges–Proskauer, methyl red, citrate reactions, gelatin liquefaction, nitrate reduction, urease test, glucose oxidation and carbohydrate fermentations were determined), Gram-negative staining, the presence of normally positive cytochrome oxidase and catalase reaction. Additionally, commercial identification kit systems API 20E and API 20NE (BioMérieux, https://www.biomerieux.com, accessed on 5 June 2021) were used. Strains were maintained on Tryptone Soya Agar (TSA) (Oxoid, Thermo Fisher Scientific, Basingstoke, UK).

### 2.4. Antimicrobial Susceptibility Testing

Susceptibility to antimicrobial agents was performed by the disk diffusion technique of Kirby–Bauer on Mueller–Hinton agar plates (Oxoid, Thermo Fisher Scientific, Basingstoke, UK) with inocula adjusted to an optical density of 0.5 McFarland standard units, according to the Clinical Laboratory Standards Institute guidelines [20]. Isolates were tested against twenty-five antibiotics, belonging to seven classes, namely beta-lactams (aminopenicillins; ureidopenicillins; monobactams; carbapenems and cephalosporins), quinolones, aminoglycosides, macrolides, amphenicols, sulfamides and phosphonic acid derivates group.

The following disks (Oxoid, Thermo Fisher Scientific, Basingstoke, UK) were used: amoxicillin (AML 10 μg), amoxicillin/clavulanic acid (AMC 30 μg), ticarcillin (TIC 75 μg), ticarcillin/clavulanic acid (TIM 85 μg), piperacillin (PRL 100 μg), piperacillin/tazobactam (TZP 110 μg), aztreonam (ATM 30 μg), imipenem (IMP 10 μg), cephalothin (KF 30 μg), cefoxitin (FOX 30 μg), ceftazidime (CAZ 30 μg), cefotaxime (CTX 30 μg), ceftriaxone (CRO 30 μg), cefoperazone (CFP 30 μg), cefepime (FEP 30 μg), nalidixic acid (NA 30 μg), ciprofloxacin (CIP 5 μg), amikacin (AK 30 μg), gentamicin (CN 10 μg), tobramycin (TOB 10 μg), kanamycin (K 30 μg), erythromycin (E 15 μg), chloramphenicol (C 30 μg), the combination sulfamethoxazole/trimethoprim (SxT 25 μg) and fosfomycin (FOS 50 μg).

Inhibition was measured after incubation at 37 °C for 18 to 24 h, and isolates were classified as susceptible, intermediate (reduced susceptibility), or resistant. *Escherichia coli* ATCC 25,922 was used as a reference strain for antibiotic disc control.

### 2.5. Biofilm Formation

The biofilm formation assay was Stepanović and colleagues’ method [21]. Briefly, overnight cultures were adjusted to an initial OD (620 nm) of 1 × 10^8^ cells/mL in Mueller–Hinton broth (MHB) and 200 µL aliquots were added to the microplate. The plate was incubated aerobically with agitation at 150 rpm and 30 °C, for 24 and 48 h. For the 48 h-old biofilms, the medium was carefully discarded and replaced by a fresh one on a daily basis. After each biofilm development period, the content of the wells was removed, and each well was washed three times with 250 μL of sterile saline solution (0.85% *v/v*) to discard non-adhered bacteria. The microtiter plate was air-dried for 30 min, and the remaining attached bacteria were analyzed in terms of biomass adhered on the surface of the microtiter plates. Wells with MHB without bacteria were used as negative controls.

### 2.6. Biomass Quantification

The biomass was quantified by crystal violet (CV) (Gram colour-staining set for microscopy, Merck, Algés, Portugal) staining according to Simões and collaborators [22]. The biofilms in the 96-well plates were fixed with 250 μL of 98% ethanol per well, for 15 min. Afterwards, the ethanol was discarded, the plates left to dry and then the fixed biofilm was stained with 200 μL of 1% CV (Merck, Algés, Portugal) for 5 min. After this, the plates were air-dried and the dye bound to the adherent cells was resolubilized by adding 200 μL of 33% (*v/v*) glacial acetic acid (VWR, Alfragide, Portugal). The optical density (OD) of each well was measured at 570 nm using an automated microtiter plate reader (Spectrostar nano, BMG Labtech, Ortenberg, Germany).

## 3. Results

**Figure 1:**
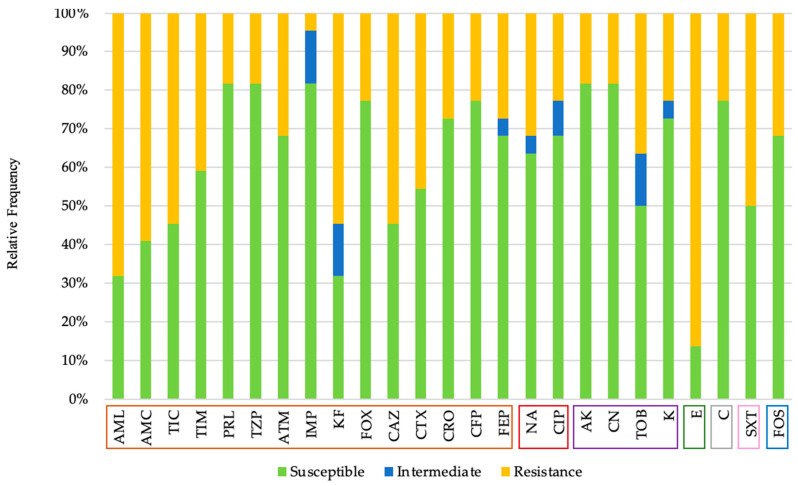
Antibiotic susceptibility profile of Gram-negative isolates, according to the following seven classes: beta-lactams (orange; Amoxicillin (AML), Amoxicillin/Clavulanic Acid (AMC), Ticarcillin (TIC), Ticarcillin/Clavulanic Acid (TIM), Piperacillin (PRL), Piperacillin/Tazobactam (TZP), Aztreonam (ATM), Imipenem (IMP), Cephalothin (KF), Cefoxitin (FOX), Ceftazidime (CAZ), Cefotaxime (CTX), Ceftriaxone (CRO), Cefoperazone (CFP), Cefepime (FEP)), quinolones (brown; Nalidixic Acid (NA), Ciprofloxacin (CIP)), aminoglycosides (violet; Amikacin (AK), Gentamicin (CN), Tobramycin (TOB), Kanamycin (K)), macrolides (green; Erythromycin (E)), amphenicols, (grey; Chloramphenicol (C)), sulfamides (pink; Sulfamethoxazole/Trimethoprim (SxT)) and phosphonic acid derivates group (blue; Fosfomycin (FOS)).

Twenty-two Gram-negative bacterial isolates were obtained from the cultures. All tested isolates demonstrated resistance to, at least, one antibiotic. It was possible to observe multidrug resistance in 11 of the 22 isolates (50%), i.e., resistance to at least one agent in three or more antimicrobial categories [23,24]. The observed resistance was 36.67% to beta-lactams, 27.27% to quinolones, 23.86% to aminoglycosides, 86.36% to macrolides, 27.73% to chloramphenicol, 50.0% to sulfamides and 31.82% to phosphonic acid derivates. It should be noted that an isolate showed phenotypic resistance to imipenem, an antibiotic only with hospital use authorization [25], in addition to a resistance profile to four different groups of antibiotics, which are beta-lactams, quinolones, macrolides, and amphenicols (Figure 1). The combination of an aminopenicillin and carboxypenicillin with a β-lactamases inhibitor was effective in reducing resistance, as shown by the decrease in the proportion of resistant strains: 68.18% (amoxicillin) versus 59.09% (amoxicillin/clavulanic acid); 54.55% (ticarcillin) versus 40.91% (ticarcillin/clavulanic acid). However, the association of a ureidopenicillin, in this case, piperacillin, with tazobactam, which is also a β-lactamases inhibitor, has not shown a decrease in resistance. Thus, piperacillin and piperacillin with tazobactam demonstrate equal resistance.

The twenty-two obtained isolates were tested for biofilm formation. Six isolates from different lemurs (ZooO1/ZooO2/ZooO3/ZooO5/ZooO7/ZooO8) were able to form biofilms (Table 1). These isolates, which formed biofilms, produced the highest biomass amount for 48 h sampling times. In general, ZooO7 produced the lowest biomass amount for 24 h and 48 h sampling times, while ZooO3 and ZooO8 produced the higher biomass amount for both time points. Isolates obtained from ZooO1, ZooO5 and ZooO2 had a similar biofilm production over time. The diverse strains were classified in terms of biofilm productivity as weak, moderate, or strong biofilm producers. ZooO3 and ZooO8 were the only two that showed a strong ability of biofilm formation, at 48 h sampling times. All the isolates showed an increasing ability of biofilm formation over time: ZooO1, ZooO2, ZooO5 and ZooO7 showed a weak ability at 24 h and a moderate ability at 48 h; ZooO3 and ZooO8 showed a moderate ability at 24 h and a strong ability at 48 h, as previously mentioned.

## 4. Discussion

The identification of multidrug-resistant bacteria in samples from the oral cavity of clinically healthy captivity lemurs, emphasized a public health problem that concerns the emergence, increasingly frequently, of multidrug-resistant microorganisms [26,27]. The oral microbiota has been the subject of several analyses and, more recently, it is also beginning to be investigated in order to detect the prevalence of antibiotic resistance genes in humans [28], due to the fact that the oral cavity is a niche with differentiating resistance characteristics. The oral cavity is characterized by containing multiple microorganisms, which are organized in biofilms or in suspension, called bacterial aerosols. However, the bacteria organized into biofilms are those that deserve the most careful attention with regard to the bacterial resistance issue. Biofilms are provided with a dynamic biological system of microbial cells that are strongly associated with a surface and embedded in an organic polymeric matrix of a microbial origin [19]. There are data that indicate that over 65% of microbial infections are caused by microorganisms that grow on biofilms [29], most likely due to the presence of a matrix of extracellular polymeric substances, low growth rate, presence of persister cells and expression of possible biofilms specific resistance genes [30,31]. Biofilm resistance is characterized by a variation from one microorganism to another, being a combination of several mechanisms strongly influenced by the environmental conditions [32,33]. The fact of being influenced by environmental conditions may help to explain the phenotypic resistance of bacteria isolated from the oral cavity, due to the fact that they have a varied diet provided by the animal’s keepers at the zoological park where they live.

Similar to companion animals, wildlife animals that reside in zoological parks can also suffer from diseases and traumatic lesions of the oral cavity. The most frequent are tooth fractures, periodontal disease, dental malocclusion, soft tissue and musculoskeletal trauma and other unspecified acquired defects [34], which are the target of treatment by the responsible veterinarian. As previously mentioned, the diet provided to these animals may have influenced the results obtained here and other studies concerning the oral cavity microbiota. The differences of food between one animal of the same species in captivity and another in the wildlife can be significant with regard to the source of the food obtained and frequency of feeding [34]. In addition, it is believed that habits in the natural environment promote more effective hygiene of the oral cavity, despite the fact that zoological parks try to match the characteristics of wildlife. Consequently, reports of dental plaque accumulation in the animals from zoos are high, which may be synonymous with a more diverse oral microbiota and, consequently, the appearance of more multidrug-resistant bacteria. Taking this into consideration and in view of the obtained results, the importance of the research in wildlife species is justified, namely regarding their oral cavity. The results obtained here also prove the importance of individual protection equipment in a variety of professional activities, namely animal’s keepers and specialists in veterinary dentistry when called to manage oral conditions in wildlife and zoo animals.

In this zoological park, the use of antibiotics in dental procedures is based on the World Small Animal Veterinary Association’s Global Dental Guidelines, which do not recommend the use of antibiotics in prophylactic interventions. Despite not being the target of successive antibiotic treatments, animals in zoological parks have been associated with the transmission of multidrug-resistant zoonotic pathogens to humans [35]. More specifically, animals—particularly wild animals—are believed to be the source of 70% of all emerging infections [36].

Ahmed and colleagues (2007), which studied mammals, birds, reptiles, and water sources, found that approximately 21% of the isolates showed multidrug resistance phenotypes and have at least one antimicrobial resistance gene [35]. Here, it was also found that many of the isolates have phenotypic antibiotic resistance which is frequently used for the treatment of serious hospital infections [37]. In contrast to what was obtained in this study, the bacterial isolates identified by Panda and colleagues (2018) were all susceptible to imipenem [38]. In turn, Smith and collaborators (2014) decided to investigate two unrelated wildlife species herring gulls (*Larus argentatus*) and a hybrid deer (*Cervus elaphus* × *Cervus nippon*) [39]. In this case, the prevalence of resistant isolates was higher in herring gulls (87%) compared to deer (31%). The analysis and comparison of these various studies suggest that resistance, once developed, is not confined to the limits of the ecological niche where it primarily emerged, with all cases having no apparent exposure to antimicrobials [26].

Despite the fact that the contact between humans and lemurs is quite reduced, the appearance of multidrug-resistant bacteria is alarming due to its zoonotic potential, as already mentioned in other studies [40]. Daily, the animal keepers enter the space to carry out the work of handling the animals and maintaining the facilities, this being the only time where contact between animals and humans is facilitated. However, this contact at certain times of the day may be enough for the transmission of pathogens and, consequently, their spread [35]. In addition, all zookeepers should be provided with personal protective equipment when daily maintaining the spaces where wild animals are located. The main critical points are the handling and cleaning of the feces and urine, biological vehicles that transmit multi-resistant bacteria [26], and the management of animals with special emphasis on the risk of accidental bites [38].

The large-scale use of beta-lactam antibiotics, considered broad-spectrum for the treatment of infectious diseases in humans and animals [41], contributed to the emergence of resistant bacteria, including bacteria from animal origin [19]. The emergence and global spread of *Enterobacteriaceae* that are resistant to carbapenems is a threat to public health, as they are associated with high rates of morbidity and mortality [42]. According to the World Health Organization 2015 report [43], in 2050, ten million annual deaths will be attributed to antimicrobial resistance.

Multidrug resistance to a variety of antibiotics was observed in this study. The various antibiotics tested here are classified as critically important (CIP, E, FOS, CN, K) for human medicine [44] and, according to the World Organization for Animal Health, categorized as veterinary critically important (AMP, CIP, E, CN, K) and veterinary highly important (FOS) antimicrobial agents [45]. There was even an isolate that demonstrated a phenotypic resistance to imipenem. Imipenem is a beta-lactam antibiotic, considered as an “antibiotic of last resort” [25] and which is used to treat multidrug-resistant bacterial infections in humans, that is, for exclusive hospital use. It was concluded that lemurs are carriers of bacteria with resistance to several antibiotics, highlighting the existence of these same dispersed resistances in ecological niches. This resistance to antibiotics in wildlife animals is highly worrying for human and animal health, as it can create a continuous selective pressure in beta-lactamases [19]. The results obtained here also alert us to the fact that the vast majority of the zoological parks are located in the centers of cities with a high population density. This raises the question of how waste generated by zoological parks is treated. If these residues are not disposed of correctly, the risk of perpetuating antimicrobial resistance increases exponentially. In this case, a chain of contamination of waters, soils, agricultural fields, and wild animals is established, with direct damage to humans, companion animals, and production animals [26].

## 5. Conclusions

Human and veterinary medicine are involved in this public health problem. The abuse of antibiotics leads to the spread of resistance genes in the environment and should be avoided and prevented. Bacterial cultures with their respective antibiogram should be increasingly privileged with the aim of treating infections correctly and only when justified. This work demonstrates the importance of conducting monitoring studies in wildlife animals, integrated in the “One Health, One Medicine, One World” concept, as these animals can be carriers of bacteria with genes resistant to various antibiotics. Only in this way will it be possible to prevent the dissemination of antibiotic resistant microorganisms and genetic determinants containing antibiotic-resistant genes.

Finally, the authors of this study are not aware that similar work has been carried out in the lemur animal species, so the main objective of this article was to perform a screening of Gram-negative bacteria in the oral cavity of *Varecia variegata,* associated with a profile of antibiotics representative of the main groups used to treat infections. In future studies, the objective will be a more detailed investigation of the antibiotics usually used in clinical practice and the Gram-negative and Gram-positive bacteria from the oral cavity of this animal species.

## Figures and Tables

**Table 1:** Description of the biofilm formation ability of the isolates (six from twenty-two) according to the classification proposed by Stepanović and colleagues. Legend: (0) OD ≤ 2 × ODc—non-biofilm producer; (+) ODc < OD ≤ 2 × ODc—weak biofilm producer; (++) 2 × ODc < OD ≤ 4 × ODc—moderate biofilm producer; (+++) 4 × ODc < OD—strong biofilm producer.

Animal (Biofilm/ /Iisolates)	24 h	48 h
ZooO1 (1/6)	+	++
ZooO2 (1/2)	+	++
ZooO3 (1/2)	++	+++
ZooO5 (1/7)	+	++
ZooO7 (1/4)	+	+
ZooO8 (1/1)	++	+++

## Data Availability

The data presented in this study are available on request from the corresponding author.
